# Founder mutations and rare disease in the Arab world

**DOI:** 10.1242/dmm.050715

**Published:** 2024-06-26

**Authors:** Dana Marafi

**Affiliations:** ^1^ Department of Pediatrics, College of Medicine, Kuwait University, P.O. Box 24923, 13110 Safat, Kuwait; ^2^ Section of Child Neurology, Department of Pediatrics, Adan Hospital, Ministry of Health, Hadiya 52700, Kuwait; ^3^ Kuwait Medical Genetics Centre, Ministry of Health, Sulaibikhat 80901, Kuwait

**Keywords:** Arab population, Founder mutations, Rare disease

## Abstract

Founder mutations are disease-causing variants that occur frequently in geographically or culturally isolated groups whose shared ancestor(s) carried the pathogenic variant. While some disease alleles may vanish from the genetic pool due to natural selection, variants with weaker effects may survive for a long time, thereby enhancing the prevalence of some rare diseases. These are predominantly autosomal recessive diseases but can also be autosomal dominant traits with late-onset or mild phenotypes. Cultural practices, such as endogamy and consanguinity, in these isolated groups lead to higher prevalence of such rare diseases compared to the rest of the population and worldwide. In this Perspective, we define population isolates and the underlying genetic mechanisms for accumulating founder mutations. We also discuss the current and potential scientific, clinical and public-health implications of studying founder mutations in population isolates around the world, with a particular focus on the Arab population.

## Teaser

**Summary:** Founder mutations account for increased prevalence of certain rare diseases within isolated groups in different populations. Here, we discuss the various implications of studying founder mutations in the Arab World.

## Definition of founder mutations

Founder mutations, also known as founder variants, are disease-causing genetic alterations observed at a high frequency in geographically or culturally isolated groups of which one or more of their shared ancestors carried the altered gene. These groups are known as genetic isolates owing to their cultural or geographic isolation over many generations, either by preference to maintain heritage, wealth, culture and/or religion, or by geographical or societal restrictions (Hatzikotoulas et al., 2014; Kurki et al., 2023; Zeggini, 2014). Their non-random mating/marital practices, known as endogamy (Box 1), result in a restricted genetic pool and genetic homogeneity. This is due to linkage disequilibrium (Box 1) combined with limited genetic inflow from co-existing subpopulations (Hatzikotoulas et al., 2014; Zeggini, 2014). In the Arab population of the Middle East, where consanguinity (Box 1) is common, this effect may be further amplified (Alkuraya, 2010; Temaj et al., 2022). These practices combined with the high carrier rate (Box 1) of common founder mutation(s) result in a higher prevalence of some rare genetic diseases compared to the rest of the population and worldwide (Xiao and Lauschke, 2021).

Detecting and characterizing founder mutations within an isolated group could greatly help understanding the disease history and genealogy, and have several implications on disease diagnosis, management, and prevention

Detecting and characterizing founder mutations within an isolated group could greatly help understanding the disease history and genealogy, and have several implications on disease diagnosis, management, and prevention. Additionally, it can enhance worldwide understanding of rare diseases.

**Box 1. Glossary**

**Ancestral chromosomal haplotype:** Chromosomal segments comprising certain variants or polymorphisms inherited together as a unit from a single ancestor.

**Autozygosity mapping:** Positional mapping strategy in which the DNA segments or haplotypes that are autozygous, i.e. inherited from the same ancestor (see Identity-by-descent), are identified by using specific genetic markers.

**Bottleneck events:** External (environmental) events, such as war, famine or disease, that reduce the size and genetic diversity of population to a more homogeneous smaller population.

**Carrier rate:** Proportion of individuals within a population carrying a single copy of a specific recessive genetic variant/mutation.

**Coefficient of inbreeding:** Probability that two alleles at any locus are identical-by-descent from a common ancestor.

**Consanguinity:** Marriage or reproductive relationship between closely related individuals, i.e. individuals who are related by descent from a common ancestor, e.g. marriage between first-degree or second-degree cousins.

**Endogamy:** Mating/marital practices that strictly occur between members of a local group based on shared characteristics such as religion, ethnicity or origin.

**Identity-by-descent (IBD):** Two identical chromosomal segments of DNA (alleles) of common ancestral origin.

**Identity-by-state (IBS):** Two identical alleles by chance alone.

**Linkage disequilibrium:** Non-random association of certain segments of DNA (alleles) at different genomic positions/loci in a certain population.

**Native American myopathy (NAM):** Autosomal recessive congenital muscle disorder (also known as Bailey-Bloch congenital myopathy) first described in the admixed Lumbee tribe of North Carolina (USA).

**Natural (negative) selection:** Disappearance or elimination of a certain allele with a harmful effect from the genetic pool due to early lethality in affected individuals or reproductive disadvantages.

**Selective advantages:** Survival of a certain allele in the genetic pool for several generations due to favorable characteristic in heterozygote carriers within particular environments, such as protection from disease.

**Variome database:** Genetic database containing the entire set of variants identified in different individuals within the population.

## Genetic mechanisms of founder mutations

The founder effect is the establishment of a new population from a small group of founding individuals with similar genetic characteristics due to external bottleneck events (Box 1) or due to internal preferences resulting in geographical and cultural isolation (Zeggini, 2014; Hatzikotoulas et al., 2014). In turn, this results in genetic drift in which the group becomes genetically distinct from its parental population with either a higher or lower allele frequency (Zeggini, 2014; Hatzikotoulas et al., 2014).

Owing to early lethality or reproductive disadvantages, some disease-causing alleles may quickly vanish from the population because of strong natural selection (Box 1) against mutations of severe effect. However, variants that carry a weaker effect can escape natural selection, persist for a long time and have the potential to enhance the prevalence of some rare genetic conditions (Quintana-Murci, 2016). This includes mutations associated with autosomal recessive diseases, which may even have a selective advantage (Box 1) for heterozygous carriers in particular environments. This is the case for sickle cell disease and thalassemia (caused by mutations of the *HBB* gene) as both confer protection against malaria, and also for lysosomal storage disease in which the partial enzyme deficiency causes cell resistance to some infectious agents (Kariuki and Williams, 2020; Fan et al., 2023; Pieroni et al., 2021). In some instances, these selective advantages for heterozygous carriers result in more than one frequent mutation of the same gene within the genetic pool of a population, raising the prevalence of the rare disease even further (Kaback, 2000). An alternative explanation for such phenomenon is the recent mixing of smaller groups or subpopulations that historically were distinct or isolated from each other but carry different founder mutations in the same disease gene (Xue et al., 2017). Less often, such surviving mutations may be associated with mild or late-onset (post-reproductive lethal) autosomal dominant diseases (Mahadevan et al., 2016).

Founder mutations underlying autosomal recessive disease traits are typically located on an ancestral chromosomal haplotype (Box 1) that is reunited in affected individuals due to identity-by-descent (Box 1) (Alkuraya, 2010). The two identical chromosomal segments are defined as autozygous because of their common ancestral origin – in contrast to being defined as homozygous, i.e. when two identical segments in the gene pool of the population are united by chance due to identity-by-state (Box 1) (Alkuraya, 2010). Autozygosity mapping (Box 1) is a powerful method for determining underlying disease mutations and estimating when the mutations arose (Alkuraya, 2010, 2013). Understanding the mechanisms of founder mutations can reveal important information about population genetics and history, and improve our ability to detect them.

## Founder mutations around the world

Founder mutations have been documented in many if not all populations and genetic isolates around the globe, and in every single continent. They can occur at a level at which an entire population can trace its roots back to a limited number of founding ancestors migrating to the region thousands of years ago. This can be due to maintenance of their cultural and socio-geographical isolation from neighboring populations/countries, such as the case with populations in Finland (Arcos-Burgos and Muenke, 2002; Uusimaa et al., 2022), Sardinia (Arcos-Burgos and Muenke, 2002), Palau Island (Arcos-Burgos and Muenke, 2002), Margarita (Arcos-Burgos and Muenke, 2002), Costa Rica (Arcos-Burgos and Muenke, 2002), Greenland (Zeggini, 2014) and Iceland (Zeggini, 2014; Hatzikotoulas et al., 2014). More commonly, it occurs in ethnohistorical, ethnoreligious and ethnolinguistic isolated groups, such as Anabaptist Christian groups in North America (e.g. Hutterites, Old Order Amish and Mennonites) (Payne et al., 2011; Zeggini, 2014; Hatzikotoulas et al., 2014; Arcos-Burgos and Muenke, 2002), Ashkenazi Jewish (Arcos-Burgos and Muenke, 2002; Slatkin, 2004), Basques of Europe (Garagnani et al., 2009), the Paisa community in Colombia (Arcos-Burgos and Muenke, 2002), Berber in North Africa (Romdhane et al., 2012), Afrikaners in South Africa (Lambie et al., 2015), Indo-Europeans (Müller et al., 2004), Austronesians (Yu et al., 2023; Hou, 2015), Cajun French in Louisiana and French-Canadians (Roy-Gagnon et al., 2011), and European Roma (Morar et al., 2004; Martínez-Cruz et al., 2016). Many of these groups have migrated because of unfavorable historical bottleneck events or fled religious prosecution and resided in new areas, segregating themselves from the rest of the population for generations. Each of these populations have their own founder mutations and have contributed to the discovery of many unique rare genetic diseases by using genetic mapping (Arcos-Burgos and Muenke, 2002). Furthermore, several genetic isolates maintain well-documented extended pedigrees, which may allow tracing the mutation back to the founder individual (Hatzikotoulas et al., 2014). Some of these rare conditions are even regarded as unique genetic disorders to the isolate group, such that they have been used for genealogical studies (Uusimaa et al., 2022; Kurki et al., 2023).

Improved equity of genetic testing would impact greatly on our understanding of rare disease around the world.

In a study of 508 autosomal recessive disorders, ∼27% were found to be limited to or enriched in specific populations (Xiao and Lauschke, 2021). In Finland, one out of every five individuals with Finnish heritage carries a variant associated with at least one Finnish Heritage Disease (Uusimaa et al., 2022). Yet, owing to global inequities in access to clinical and research genetic testing, the global prevalence of founder mutations is likely to be underestimated. For example, relatively few founder mutations have been reported in Latinos, indigenous populations in America that were shaped by bottleneck and founder events (Belbin et al., 2018; Gonzaga-Jauregui et al., 2015). Similarly, the homozygous *STAC3* variant (c.851G>C; p.Trp284Ser) responsible for Native American myopathy (Box 1) has only recently been recognized to have an African origin because of the growth of African genomics (Raga et al., 2022). Improved equity of genetic testing would impact greatly on our understanding of rare disease around the world.

## Structure of the Arab population in the Middle East

**Fig. 1.**
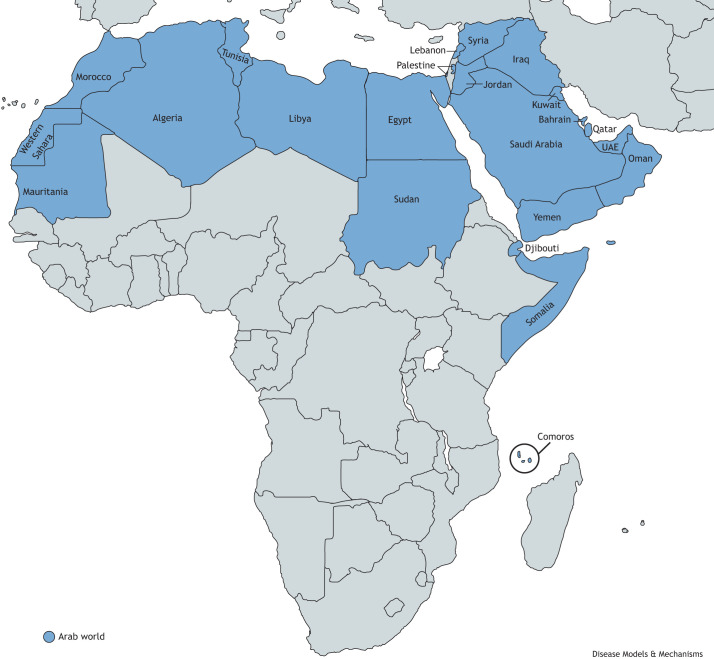
Map showing the Arab nations of the modern Middle East.

The Arab population is an ethnolinguistic group defined by their shared culture, spoken language and heritage. The Arab population is mostly tribal (Al-Gazali et al., 2006; Al-Owain et al., 2012). Arab tribes are historically nomadic Bedouins who originally inhabited the Arabian Peninsula, and later spread to the Levant region (Syria, Lebanon, Jordan, Palestine), North Africa (Egypt, Sudan, Libya, Tunisia, Algeria, Morrocco, Mauritania), Djibouti, Comoros and Somalia, which represent the Arab nations of the modern Middle East (Fig. 1) (Zayed, 2016). Present-time Arab tribes trace their roots back to two founding genealogical linages, the Adnanite (General and west Eurasian Arab) or Qahtanite (Peninsular Arab) lineages named, respectively, after their two Arab forefathers Adnan or Qahtan (Razali et al., 2021; Mohammad et al., 2009). Despite nationalization of the Bedouin society by the governments of Middle East countries and the abandonment of nomadism, tribalism still exists. However, members of a single tribe might reside in several neighboring countries, depending on their historical geographic land and, so, they do not necessarily confine to geographic boundaries of one country (Al-Gazali et al., 2006). Many tribes remain in social isolation, with strict mating/marital patterns despite socially intermixing with other groups and tribes in Arab countries of the modern era (Al-Gazali et al., 2006). Thus, the population of Arab countries is composed of several subpopulations of internal isolates (Al-Gazali et al., 2006; Al-Owain et al., 2012; Arcos-Burgos and Muenke, 2002). Intra-tribal unions are very common, in some regions reaching up to 90%, and can even involve intra-familial consanguineous marriages (Nabulsi, 1995). Inter-tribal unions also commonly occur but are often strictly guided by inter-tribal relations, such as historical alliances, geographical proximity or shared ancestry between these tribes. Consistent with these cultural practices, a study from Saudi Arabia found that tribalism influences genetic structure, such that genetic clustering occurs within tribes in individuals who self-identify with 28 large Arabian tribes (Mineta et al., 2021). These individuals had a high coefficient of inbreeding (Box 1) at its highest in tribes with historically strict intra-tribal marriages (Mineta et al., 2021). Furthermore, the distance between the genetic clusters correlates with the geographic proximities of these tribes in Saudi Arabia, which enables tribal intermarriage and admixture (Mineta et al., 2021). Although many founder mutations are tribe-specific or even branch-specific within a certain tribe, several multi-tribe founder mutations have either been observed in multiple Arab countries – so-called ‘Ancient Arab founder mutations’ that serve as evidence of Arab tribes co-ancestry – or within a certain region indicating recent admixture between tribes (Al-Owain et al., 2012; Mineta et al., 2021; Markus et al., 2014). The unique characteristics of the Arab population has resulted in many unique rare disease discoveries.

## Rare disease and founder mutations in Arab populations

Over 160 rare genetic syndromes were first described in Arab populations (Teebi, 1997). Many of these syndromes, such as Sanjad-Sakati syndrome, Woodhouse-Sakati syndrome and Temtamy syndrome, occur almost exclusively in Arabs and have been attributed to ancient founder variants in Arabs (Sanjad et al., 1991; Parvari et al., 2002; Temtamy et al., 1996; Zahrani et al., 2013; Alrakaf et al., 2018; Kohil et al., 2023). Founder mutations identified in the Arab region have also contributed to several new disease–gene associations. Shaheen syndrome was discovered after identifying a deep intronic tribal founder variant in three Saudi families with intellectual disability, hypohidrosis and microcephaly (Shamseldin et al., 2023; Shaheen et al., 2013). Similarly, a Saudi founder variant of *DBR1* is the underlying reason for this gene being associated with a lethal form of congenital ichthyosis (Musa et al., 2019; Alrohaif et al., 2018). Some of these disease-gene discoveries facilitated by investigating Arab founder mutations have been later replicated in other populations, giving rise to a broader phenotype spectrum than that of the fairly homogenous phenotype associated with the original founder variant (Kono et al., 2009). In parallel, Arab founder mutations have also confirmed the candidacy of previously identified disease genes and improved our understanding of many existing rare diseases, such as mitochondrial MICU1 deficiency and GNE myopathy (Musa et al., 2019; Alrohaif et al., 2018). Table 1 lists a selected number of relatively common ancient Arab founder mutations and their associated rare diseases/syndromes (Tadmouri et al., 2006).

**Table 1.**
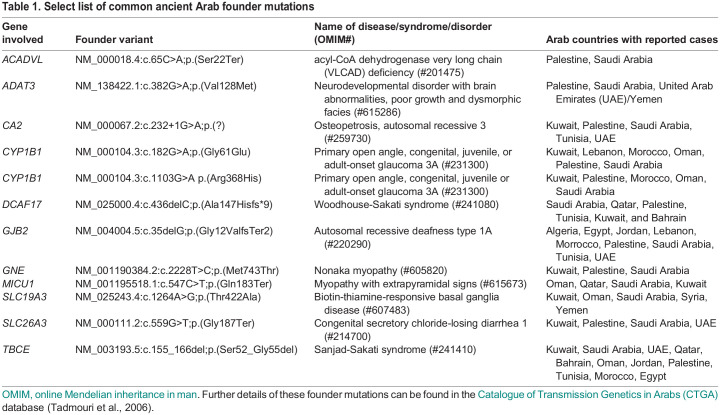
Select list of common ancient Arab founder mutations

Occasionally, the Arab founder variant, such as that in *ADAT3* (El-Hattab et al., 2016), *C12ORF57* (Alrakaf et al., 2018), *DCAF17* (Kohil et al., 2023), *GNE* (Sela et al., 2013) and *RUBCN* (Seidahmed et al., 2020), may be the only or the main variant described to cause the disease. Furthermore, a single founder mutation may be the most common genetic etiology underlying a specific disease phenotype in the region. For instance, an Arab multi-tribal founder variant in *ADAT3* was found to be the most common variant in autosomal recessive intellectual disability in Saudi Arabia, accounting for <1% of all cases undergoing exome sequencing (Monies et al., 2019).

Although ancient founder mutations have been reported in several Arab countries (Table 1), many are well-recognized to be tribe-specific, such as a *DNAI2* tribal founder variant implicated in primary ciliary dyskinesia in Kuwait and a *COG6* Saudi founder variant associated with Shaheen syndrome (Shaheen et al., 2013; Al-Mutairi et al., 2022). Although data on the prevalence of founder mutations in rare genetic diseases in Arabs are sparse, studies from Saudi Arabia have shown that 30-40% of detected variants in all genetic cases are caused by a founder variant (Monies et al., 2017, 2019). These observations have been replicated in several disease-specific cohorts, including ciliopathies and skeletal dysplasia (Shaheen et al., 2016; Maddirevula et al., 2018). Information on prevalence of founder mutations in other populations are quite limited as it requires large-scale genetic studies, and genetic databases that are extensive and comprise ethnically diverse population data in order to define the founder mutations within the population and estimate their burden.

Interestingly, Middle Eastern founder mutations have also been described in cancer. Two Middle Eastern founder variants in *BRCA1* account for 46% of all *BRCA* mutant cases and 1.6% of all breast cancer cases in a small Middle Eastern cohort (Bu et al., 2016). Therefore, these founder mutations have a clear impact on human health and disease.

## Clinical implications of founder mutations

**Fig. 2.**
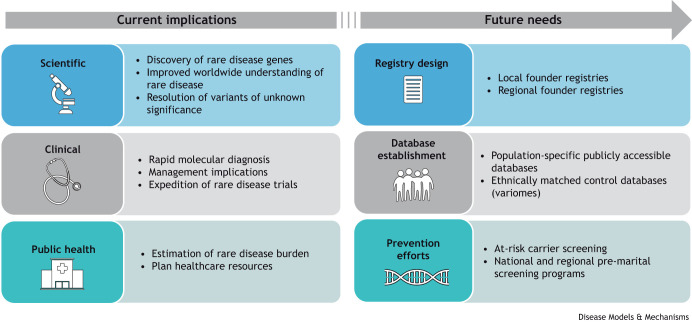
Current and future scientific, clinical and public-health implications of studying founder mutations in population isolates around the world.

Understanding predominant founder mutations within a population offers great benefits for the scientific field and society (Fig. 2). Knowledge of the existence of certain rare diseases within an isolated tribe or community not only allows rapid clinical diagnosis and molecular confirmation through targeted genetic testing but also may alleviate the need for more invasive clinical testing, such as liver or muscle biopsy (Al-Owain et al., 2012). Targeted genetic testing is more cost effective and has rapid turnaround time because of reduced analysis complexity compared to more-extensive unbiased genetic testing with next-generation sequencing of exomes or genomes. In some instances, founder mutations can be deeply intronic and, thus, might not be captured with several first-tier exome sequencing, requiring either strong clinical suspicion for targeted testing or gene sequencing, or more-sophisticated testing, such as RNA or genome sequencing (Khan et al., 2017). Founder mutations have also been shown to help resolve variants of unknown significance in different populations (Gallione et al., 2011).

Additionally, early clinical suspicion of a specific disease can facilitate initiation of precise treatment and guide management. For example, for biotin- or thiamine-responsive encephalopathy type 2 (also known as biotin-thiamine responsive basal ganglia disease, see Table 1) that is associated with an Arab founder variant in *SLC19A3*, rapid clinical and molecular diagnosis together with early initiation of high-dose biotin and thiamine not only prevent long-term sequela but can result in complete reversal of neuroradiological findings and restoration of normal baseline neurological function (Aburezq et al., 2023). Similarly, patients carrying founder mutations in *OSTM1*, the underlying cause of the majority of cases of malignant infantile osteopetrosis (MIOP) in Kuwait, are not typically offered invasive bone marrow transplantation, which is in contrast to other forms of MIOP (Alotaibi et al., 2023; Ott et al., 2013). Instead, given the severe neurological deterioration associated and early mortality with this particular form of MIOP (Alotaibi et al., 2023; Ott et al., 2013), clinicians can focus on more palliative care for these individuals.

The presence of relatively high numbers of cases due to founder mutations also enables efficient acquisition of rare disease natural history data, and facilitates enrolment in clinical trials and biomarker identification for rare disease. Clinical trials undergo several phases before launching to FDA approval and marketing; the speed of this process depends on the constant flow of patients to boost the study power, which is considered a major challenge in drug development for rare diseases (Pizzamiglio et al., 2022). The high number of available therapeutic options and ongoing gene-therapy clinical trials for Tay-Sachs disease (TSD) may be attributed, in part, to the high prevalence of TSD in certain communities, such as the Ashkenazi Jewish and Eastern Europeans, due to founder mutations (Picache et al., 2022). Thus, to expedite patient recruitment, future efforts in rare disease clinical trials should focus on connecting with worldwide centers in areas with a high prevalence of certain rare disease.

## Challenges in identifying founder mutations

When a variant has been observed repeatedly in one or different regions of the world, two possibilities exist: either it is a recurrent variant due to a mutational hotspot or it is a founder mutation (Nesta et al., 2021). Once a founder mutation is suspected based on shared ancestry, ethnic origin or tribe, or geographic proximity between the carrier individuals, confirmation can be sought by using one of three methods: haplotype analysis/autozygosity mapping, genealogical studies, or by checking for the presence of unaffected heterozygous carriers – with minor allele frequency (MAF) value of >0% – in an ethnically matched control database (Peltonen et al., 2000; Alkuraya, 2010; Ammous et al., 2021; Jaakkola et al., 2010). The length of the shared haplotype segment harboring the founder mutation can determine the age of the mutation, and differentiate between ancient co-ancestry and recent admixtures between across multiple tribes (Markus et al., 2014; Alkuraya, 2010). Limited access to research-based studies for further genetic analysis and limited numbers of ethnically matched variome databases (Box 1) for MAF calculations pose a key challenge in confirming a mutation as a founder within the population (Mineta et al., 2021). Owing to better ethnic representation and presence of unaffected heterozygous carriers within the population, a MAF value of >0 is expected to be observed more in local/ethnically matched databases than in a global database.

Another challenge is that many pathogenic variants found in the literature are either without a declared ethnicity of the subject(s) or are broadly stated as ‘Arab patient’, ‘Bedouin’, ‘Middle Eastern’ or ‘Mediterranean’ such that, when repeatedly observed, makes it hard to be flagged as a possible founder mutation in a certain tribe or a country. Yet, knowledge of tribal origin can certainly be helpful in the clinical setting (Al-Owain et al., 2012). In Arab practice, the tribal affiliation is best determined by surname, which is inherited patrilineally. A study found that the self-identified tribal affiliation of an individual aligns well with their place in the genetic cluster along the tribal line (Mineta et al., 2021). While disclosing the name of the tribe in scientific publications is guarded by social and ethical restrictions, stating the country of origin or ancestry of an individual is informative and scientifically appropriate. Thus, information pertaining to tribe name and specific village should be accessed with specific permission only. Stating the ancestral country of origin is particularly important for Arabs in conflict-affected areas and for Arab diaspora residing in countries not aligned with their ancestral country of origin (e.g. Palestinian, Iraqi, Sudanese, Syrian, Yemeni Arab diaspora) (Khaled et al., 2023). In such cases, when the same variant is again observed in another patient from the same ethnicity and ancestral country of origin, it would be appropriate to flag it as a potential founder variant in that country for further studies. Recognizing and classifying founder mutations within a population needs to be treated with cultural sensitivity, and requires proper understanding of the population stratification, tribal structure and mating behavior.

## Future directions regarding rare diseases in the Arab world

Knowledge of founder mutations within a population or a community should be harnessed to facilitate diagnosis of unsolved rare disease cases and to reduce the disease burden within a community. Several gaps in this area still exist (Fig. 2). To address these gaps, efforts should initially focus on establishing local and regional case registries, and ethnically matched control databases, as well as creating publicly accessible founder databases to catalog such mutations (Al-Owain et al., 2012; Mineta et al., 2021; Zayed, 2016). The Greater Middle Eastern (GME) Variome was initiated in 2016 as a control database for genetic variants in Arab and other Middle Eastern populations (Scott et al., 2016). Another comprehensive resource for genetic variants driven from exomes of Arab, Middle Eastern and North African populations – including the GME database as well as Qatari and Iranian exomes – is the al mena database (https://clingen.igib.res.in/almena/) established in 2017 (Koshy et al., 2017).

In last two decades there have also been continuous efforts to publish founder mutations, estimate their burden and catalog all pathogenic variants described in Arab individuals (Abouelhoda et al., 2016; Aleissa et al., 2022). The first curated catalog was called the Arab Genetic Disease Database (AGDDB) (Teebi et al., 2002). This initiative was the seed for a more-systemic organized effort, yielding the Catalogue of Transmission Genetics in Arabs (CTGA) database (https://cags.org.ae/en/search-database) (Tadmouri et al., 2006). CTGA is a publicly available database that continuously includes pathogenic mutations identified in Arab populations, some of which had been identified in several Arab countries but have not been flagged or confirmed as founder mutations. Disease Allele in Arabs (DALIA) is another comprehensive manually curated resource for disease gene variants in Arabs, and includes global and population-specific MAF, and *in silico* prediction data for clinical genetic and epidemiological use (Vatsyayan et al., 2021). An Arab genetic database specifically for founder mutations – similar to the Amish, Mennonite, and Hutterite Genetic Disorder Database (https://www.biochemgenetics.ca/plainpeople/) (Payne et al., 2011) – could be clinically useful if it enables clinicians to search by phenotypic features.

Additionally, entering and flagging unpublished founder mutations – suspected or confirmed – in individuals within a certain region into publicly available archives, such as ClinVar or GeneMatcher/VariantMatcher, can help the international community to interpret variants that, otherwise, would only be classified as variants of uncertain significance (VUS). Declaring the ethnicity of a patient may also be helpful when a variant is observed again in the same ethnicity but in a different laboratory.

The presence of a single (or a few) predominant founder mutation(s) accounting for a disease in a population provides a powerful opportunity for primary preventative measures through reproductive carrier screening. The success story of reducing the incidence of TSD by 90% after using targeted carrier screening within the Ashkenazi Jewish community is a model to follow (Kaback, 2000). The implementation of national premarital genomic screening programs in Arab countries could reduce the high burden of rare genetic disorders due to founder mutations (Delatycki et al., 2020). Such an approach should be implemented with a single genotyping assay offered indiscriminately at population-wide level, regardless of tribal affiliation, given the increasing admixture within the society. Although this approach would not eliminate the risk of rare autosomal recessive diseases caused by non-founder variants or rare founder variants not included in the screening program, it is likely to reduce the healthcare burden caused by the more common founder mutations in the country. Prior to proceeding with their endogamous and/or consanguineous marriage, couples should be offered precise counseling regarding the significant residual risk to avoid false reassurance.

In conclusion, founder mutations are common in the Arab world and worldwide, and have contributed to our understanding of rare diseases. While there are many scientific, clinical and public-health implications for their identification, addressing the significant knowledge gaps will improve their potential use in disease prevention and therapeutics.
